# Retrospective evaluation of national MRI reporting quality for lateral lymph nodes in rectal cancer patients and concordance with prospective re-evaluation following additional training

**DOI:** 10.1186/s13244-022-01303-7

**Published:** 2022-10-20

**Authors:** Tania C. Sluckin, Sanne-Marije J. A. Hazen, Karin Horsthuis, Regina G. H. Beets-Tan, Corrie A. M. Marijnen, Pieter J. Tanis, Miranda Kusters, The Dutch Snapshot Research Group, Arend G. J. Aalbers, Susanna M. van Aalten, Femke J. Amelung, Marjolein Ankersmit, Imogeen E. Antonisse, Jesse F. Ashruf, Tjeerd S. Aukema, Henk Avenarius, Renu R. Bahadoer, Frans C. H. Bakers, Ilsalien S. Bakker, Fleur Bangert, Renée M. Barendse, Heleen M. D. Beekhuis, Geerard L. Beets, Willem A. Bemelman, Maaike Berbée, Shira H. de Bie, Robert H. C. Bisschops, Robin D. Blok, Liselotte W. van Bockel, Anniek H. Boer, Frank C. den Boer, Evert-Jan G. Boerma, Leonora S. F. Boogerd, Jaap Borstlap, Wernard A. A. Borstlap, Johanna E. Bouwman, Sicco J. Braak, Manon N. G. J. A. Braat, Jennifer Bradshaw, Amarins T. A. Brandsma, Vivian van Breest Smallenburg, Wim T. van den Broek, Sjirk W. van der Burg, Jacobus W. A. Burger, Thijs A. Burghgraef, David W. G. ten Cate, Heleen M. Ceha, Jeltsje S. Cnossen, Robert R. J. Coebergh van den Braak, Esther C. J. Consten, Maaike Corver, Rogier M. P. H. Crolla, Sam Curutchet, Alette W. Daniëls-Gooszen, Paul H. P. Davids, Emmelie N. Dekker, Jan Willem T. Dekker, Ahmet Demirkiran, Tyche Derksen, Arjen L. Diederik, Anne M. Dinaux, Kemal Dogan, Ilse M. van Dop, Kitty E. Droogh-de Greve, Hanneke M. H. Duijsens, Michalda S. Dunker, Johan Duyck, Eino B. van Duyn, Laurentine S. E. van Egdom, Bram Eijlers, Youssef El-Massoudi, Saskia van Elderen, Anouk M. L. H. Emmen, Marc Engelbrecht, Anne C. van Erp, Jeroen A. van Essen, Hans F. J. Fabry, Thomas Fassaert, Eline A. Feitsma, Shirin S. Feshtali, Bas Frietman, Edgar J. B. Furnée, Anne M. van Geel, Elisabeth D. Geijsen, Nanette van Geloven, Michael F. Gerhards, Hugo Gielkens, Renza A. H. van Gils, Lucas Goense, Marc J. P. M. Govaert, Wilhelmina M. U. van Grevenstein, E. Joline de Groof, Irene de Groot, Robbert J. de Haas, Nadia A. G. Hakkenbrak, Mariska D.den Hartogh, Vera Heesink, Joost T. Heikens, Ellen M. Hendriksen, Sjoerd van den Hoek, Erik J. R. J. van der Hoeven, Christiaan Hoff, Anna Hogewoning, Cornelis R. C. Hogewoning, Stefan Hoogendoorn, Francois van Hoorn, René L. van der Hul, Rieke van Hulst, Farshad Imani, Bas Inberg, Martijn P. W. Intven, Pedro Janssen, Chris E. J. de Jong, Jacoline Jonkers, Daniela Jou-Valencia, Bas Keizers, Stijn H. J. Ketelaers, Eva Knöps, Sebastiaan van Koeverden, Sylvia Kok, Stephanie E. M. Kolderman, Fleur I. de Korte, Robert T. J. Kortekaas, Julie C. Korving, Ingrid M. Koster, Jasenko Krdzalic, Pepijn Krielen, Leonard F. Kroese, Eveline J. T. Krul, Derk H. H. Lahuis, Bas Lamme, An A. G. van Landeghem, Jeroen W. A. Leijtens, Mathilde M. Leseman-Hoogenboom, Manou S. de Lijster, Martijn S. Marsman, Milou. H. Martens, Ilse Masselink, Wout van der Meij, Philip Meijnen, Jarno Melenhorst, Dietrich J. L. de Mey, Julia Moelker-Galuzina, Linda Morsink, Erik J. Mulder, Karin Muller, Gijsbert D. Musters, Joost Nederend, Peter A. Neijenhuis, Lindsey C. F. de Nes, Mandy Nielen, Jan B. J. van den Nieuwboer, Jonanne F. Nieuwenhuis, Joost Nonner, Bo J. Noordman, Stefi Nordkamp, Pim B. Olthof, Steven J. Oosterling, Daan Ootes, Vera Oppedijk, Pieter Ott, Ida Paulusma, Koen C. M. J. Peeters, Ilona T. A. Pereboom, Jan Peringa, Zoë Pironet, Joost D. J. Plate, Fatih Polat, Ingrid G. M. Poodt, Lisanne A. E. Posma, Jeroen F. Prette, Bareld B. Pultrum, Seyed M. Qaderi, Jan M. van Rees, Rutger-Jan Renger, Anouk J. M. Rombouts, Lodewijk J. Roosen, Ellen A. Roskott-ten Brinke, Joost Rothbarth, Dennis B. Rouw, Tom Rozema, Heidi Rütten, Harm J. T. Rutten, Marit E. van der Sande, Boudewijn E. Schaafsma, Renske A. Schasfoort, Merel M. Scheurkogel, Arjan P. Schouten van der Velden, Wilhelmina H. Schreurs, Puck M. E. Schuivens, Colin Sietses, Petra C. G. Simons, Marjan J. Slob, Gerrit D. Slooter, Martsje van der Sluis, Bo P. Smalbroek, Anke B. Smits, Ernst J. Spillenaar-Bilgen, Patty H. Spruit, Tanja C. Stam, Jaap Stoker, Aaldert K. Talsma, Sofieke J. D. Temmink, G. Y. Mireille The, Jeroen A. W. Tielbeek, Aukje A. J. M. van Tilborg, Fiek van Tilborg, Dorothée van Trier, Jurriaan B. Tuynman, Maxime J. M. van der Valk, Inge J. S. Vanhooymissen, G. Boudewijn C. Vasbinder, Cornelis J. Veeken, Laura A. Velema, Anthony W. H. van de Ven, Emiel G. G. Verdaasdonk, Wouter M. Verduin, Tim Verhagen, Paul M. Verheijen, Maarten Vermaas, An-Sofie E. Verrijssen, Anna V. D. Verschuur, Harmke Verwoerd-van Schaik, Roy F. A. Vliegen, Sophie Voets, F. Jeroen Vogelaar, Clementine L. A. Vogelij, Johanna Vos-Westerman, Marianne de Vries, Joy C. Vroemen, Bas S. T. van Vugt, Johannes A. Wegdam, Bob J. van Wely, Marinke Westerterp, Paul P. van Westerveld, Henderik L. van Westreenen, Allard G. Wijma, Johannes H. W. de Wilt, Bart W. K. de Wit, Fennie Wit, Karlijn Woensdregt, Victor van Woerden, Floor S. W. van der Wolf, Sander van der Wolk, Johannes M. Wybenga, Edwin S. van der Zaag, Bobby Zamaray, Herman J. A. Zandvoort, Dennis van der Zee, Annette Zeilstra, Kang J. Zheng, David D. E. Zimmerman, Marcel Zorgdrager

**Affiliations:** 1 Department of Surgery, Amsterdam UMC Location Vrije Universiteit Amsterdam, De Boelelaan 1117, Amsterdam, The Netherlands; 2 Cancer Center Amsterdam, Treatment and Quality of Life, Amsterdam, The Netherlands; 3 Cancer Center Amsterdam, Imaging and Biomarkers, Amsterdam, The Netherlands; 4 Department of Radiology, Amsterdam UMC Location Vrije Universiteit Amsterdam, De Boelelaan 1117, Amsterdam, The Netherlands; 5 Department of Radiology, The Netherlands Cancer Institute, Plesmanlaan 121, Amsterdam, The Netherlands; 6 GROW School for Oncology and Developmental Biology, University of Maastricht, Universiteitssingel 40, Maastricht, The Netherlands; 7 Department of Radiology, Department of Clinical Research, Odense University Hospital, University of Southern Denmark, Campusvej 55, 5230 Odense, Denmark; 8 Department of Radiation Oncology, LUMC, Albinusdreef 2, Leiden, The Netherlands; 9 Department of Radiation Oncology, The Netherlands Cancer Institute, Plesmanlaan 121, Amsterdam, The Netherlands; 10 Department of Surgery, Amsterdam UMC Location University of Amsterdam, Meibergdreef 9, Amsterdam, The Netherlands; 11 Department of Surgical Oncology and Gastrointestinal Surgery, Erasmus MC, Rotterdam, The Netherlands

**Keywords:** Rectal cancer, Lateral lymph nodes, MR imaging

## Abstract

**Objectives:**

The presence and size of lateral lymph nodes (LLNs) are important factors influencing treatment decisions for rectal cancer. Awareness of the clinical relevance and describing LLNs in MRI reports is therefore essential. This study assessed whether LLNs were mentioned in primary MRI reports at a national level and investigated the concordance with standardised re-review.

**Methods:**

This national, retrospective, cross-sectional cohort study included 1096 patients from 60 hospitals treated in 2016 for primary cT3-4 rectal cancer ≤ 8 cm from the anorectal junction. Abdominal radiologists re-reviewed all MR images following a 2-h training regarding LLNs.

**Results:**

Re-review of MR images identified that 41.0% of enlarged (≥ 7 mm) LLNs were not mentioned in primary MRI reports. A contradictory anatomical location was stated for 73.2% of all LLNs and a different size (≥/< 7 mm) for 41.7%. In total, 49.4% of  all cases did not mention LLNs in primary MRI reports. Reporting LLNs was associated with stage (cT3N0 44.3%, T3N+/T4 52.8%, *p* = 0.013), cN stage (*N*0 44.1%, *N*1 48.6%, *N*2 59.5%, *p* < 0.001), hospital type (non-teaching 34.6%, teaching 52.2%, academic 53.2% *p* = 0.006) and annual rectal cancer resection volumes (low 34.8%, medium 47.7%, high 57.3% *p* < 0.001). For LLNs present according to original MRI reports (*n* = 226), 64.2% also mentioned a short-axis size, 52.7% an anatomical location and 25.2% whether it was deemed suspicious.

**Conclusions:**

Almost half of the primary MRI reports for rectal cancer patients treated in the Netherlands in 2016 did not mention LLNs. A significant portion of enlarged LLNs identified during re-review were also not mentioned originally, with considerable discrepancies for location and size. These results imply insufficient awareness and indicate the need for templates, education and training.

## Key points

- 50.6% of primary MRI reports for cT3-4 rectal cancer patients mentioned LLNs.
- Size, location, malignant features and suspiciousness were mentioned in 23–64% of reports.
- 41% of enlarged (≥ 7 mm) LLNs identified via re-review were not mentioned in the original MRI reports.

## Introduction

The comprehensive treatment of locally advanced rectal cancer (at least cT3 stage), including neoadjuvant (chemo)radiotherapy and high-quality standardised surgery according to the principles of total mesorectal excision (TME), has decreased the chance of developing a locoregional recurrence (LR) to 5–10% [1, 2]. Recent studies have suggested that one of the underlying causes of LR in low rectal cancer may be the presence of malignant lateral lymph nodes (LLNs) [3, 4]. Adequate preoperative identification of LLNs is therefore essential in order to determine an optimal treatment strategy.

Magnetic resonance imaging (MRI) is the primary modality for staging rectal cancer and is also ideal for the identification of LLNs, which are situated outside of the mesorectal fascia [5–7]. Research has established that the short-axis size and anatomical location are important factors when considering whether LLNs are suspicious for malignancy [8–10]. Various studies evaluating LLNs on the staging MRI have indicated short-axis sizes of 5–8 mm to increase the LR rate to 30–40% [11–14]. A recent international retrospective cohort study with MRI re-review revealed that a short-axis size of ≥ 7 mm increased the lateral LR (LLR) rate to 19.5% [15]. In this study, after neoadjuvant therapy, LLNs which remained enlarged (> 4 mm for internal iliac- and > 6 mm for obturator nodes) increased the LLR risk even further, with rates as high as 52.3% after 5 years [16]. Moreover, LLNs located in the internal iliac compartment appeared to reflect more aggressive disease biology, were less likely to shrink after neoadjuvant therapy and were associated with the highest LR rates [8, 16, 17], while contradictory findings have been reported regarding the prognostic impact of malignant features of LLNs [18].

Though international guidelines are currently lacking, surgeons are increasingly adhering to the notion that patients with rectal cancer and primarily enlarged LLNs, or nodes which do not respond adequately to neoadjuvant treatment, benefit from a lateral lymph node dissection (LLND). During this procedure, all lymphatic tissue lateral to the mesorectal fascia in the internal iliac and obturator compartments is removed simultaneously with TME. While this procedure has often been performed prophylactically in Japanese centres for all patients with locally advanced rectal cancer, Western hospitals have until recently been reluctant, believing neoadjuvant irradiation to be sufficient in sterilising the lateral compartments [19]. Recent research has suggested that an LLND significantly reduces the LR rates [15, 16, 20–22]. However, the oncological benefit should be weighed against associated morbidity such as nerve- and/or vascular damage, emphasising the need for careful patient selection [23, 24]. This reiterates why it is so essential that LLNs should always be mentioned in MRI reports; surgeons may advise an LLND if LLNs are present, and radiation oncologists often rely on reports to plan the appropriate delineation of the lateral compartments.

It was hypothesised that there is insufficient awareness amongst radiologists for the reporting of LLNs in routine daily practice. Therefore, the objective of this study was to evaluate how often primary MRI reports mentioned LLNs and their characteristics for patients treated for cT3-4 rectal cancer ≤ 8 cm from the anorectal junction (ARJ) in 2016 in the Netherlands. The concordance with re-review of the images by trained abdominal consultant radiologists with a focus on enlarged (≥ 7 mm) LLNs, as well as the association with the occurrence of LR, were determined.

## Methods

### Design

**Fig. 1:**
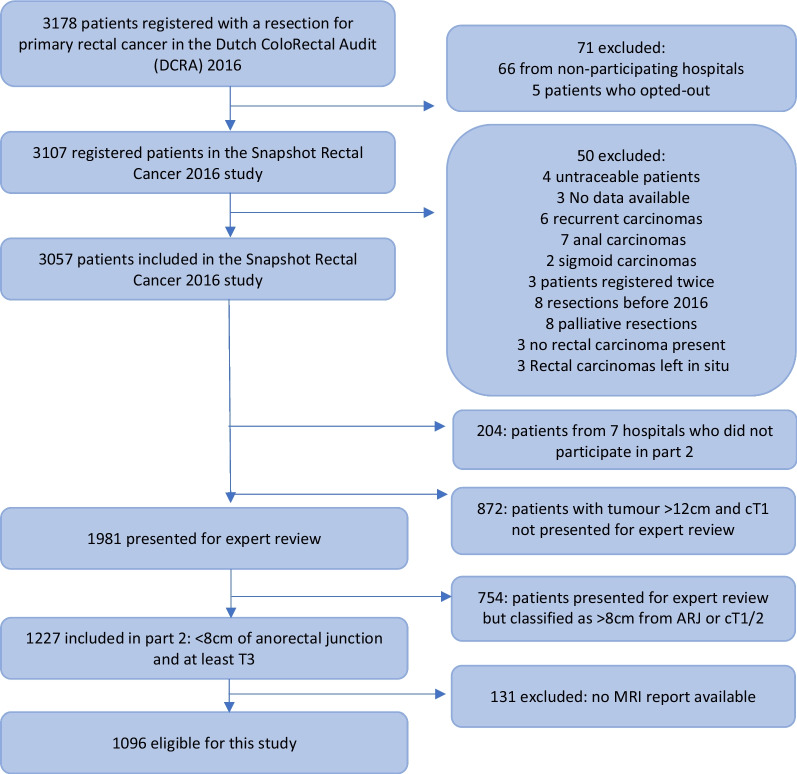
Flowchart of patient inclusion

This was a national, retrospective cross-sectional cohort study. The ‘Snapshot’ design allowed for the compilation of a large population-based data set using the principles of collaborative research in a short amount of time. A more detailed explanation can be found in a previous ‘Snapshot’ article [25]. Baseline data and short-term oncological outcomes regarding all patients treated for rectal cancer in the Netherlands are registered in the Dutch ColoRectal Audit (DCRA). The current study expanded the available data in the DCRA for 3107 of the 3178 potentially eligible consecutive patients treated for primary rectal cancer in the Netherlands in 2016 (Fig. 1).

The local collaborative research team from each centre consisted of a surgeon with supervised surgical residents, a radiologist and, if applicable, a radiation oncologist. In total, 67 of 69 Dutch hospitals in the Netherlands that provided rectal cancer care in 2016 participated. Data collection consisted of three parts, of which the first two parts are relevant to the present study. In part 1, the surgical team collected additional baseline characteristics, procedural data, and short- and long-term oncological and surgical outcomes. In part 2, 60 of the 67 centres participated, during which abdominal radiology consultants re-reviewed primary and restaging MR images.

### Data management

The project data were processed and stored anonymously by Medical Research Data Management (MRDM, Deventer, the Netherlands). MRDM is responsible for the data processing of the DCRA and is NEN7510 and ISO27001 certified.

The local surgical team only had access to data for patients in their centre within part 1. Once part 1 was completed, MRDM imported eligible patients to part 2 in a completely separate data collection location. This meant that local collaborators could not access any information about their patients outside their specific part or centre. The coordinating team received fully anonymised data: dates of birth were provided as a year of birth. All other dates, such as the date of primary MRI, had a possible 10-day spread to minimise any risk of a breach of privacy.

### Pre-assessment training

The local consultant abdominal radiologist from each centre, responsible for the MRI re-review, participated in a 2-h online training with pre- and post-training assessments. This training was provided before the start of data collection by two expert radiologists (K.H. and R.B.T. with 17 and 24 years of experience, respectively) specialised in rectal cancer and LLNs. The borders of the lateral compartments, in accordance with the colour atlas of Ogura et al. [16], were discussed, and consensus was reached to use these during the study as a guide for anatomical compartment classification. Radiologists completed a short assessment of three MRI cases before the training. This was repeated after the training with an additional three cases. The post-training assessment demonstrated an improvement in size measurements and anatomical location classification compared to pre-training assessment [26].

### Patient selection and MRI re-review

After the completion of part 1, the trained radiologists were asked to identify their target population for MRI re-review. In order not to miss eligible patients, a broader selection of patients based on data from part 1 (≤ 12 cm from the ARJ, ≥ cT2) was proffered to the radiologists for each centre separately. Radiologists then re-evaluated these factors on MRI and selected the patients with a tumour ≤ 8 cm from the ARJ and at least a clinical T3 stage for inclusion in part 2. Patients had to have an MRI report available for re-review to qualify for final inclusion (Fig. 1).

Radiologists were asked to report on all present LLNs. If present, all characteristics such as their short-axis size, anatomical location, malignant features (heterogeneity, irregular border, round shape and loss of fatty centre) and whether suspicious, were recorded. Re-review of the primary MRI occurred for all patients. If a restaging MRI after neoadjuvant treatment, or an MRI performed for a (lateral) local recurrence, was present, then these were also reviewed. Radiologists examined the T2 series with a minimum axial field of view of 150 mm, including the lateral pelvic compartments in full. The radiologists reported per patient whether a DWI was used and all original reports were submitted anonymously for central review.

### Assessment of original MRI reports

The central coordinating researchers determined whether the presence or absence of LLNs was mentioned in the primary MRI reports. A list of predetermined terms was created to ensure unambiguity (see “Appendix A”). Ambiguous terms were discussed and if it remained unclear what was meant (mesorectal or lateral), then these were not considered as LLNs. If an LLN was reported as present, then all accompanying information was extracted from the report, such as size and anatomical location as well as the presence or absence of malignant features and suspiciousness. The amount and combination of characteristics mentioned was also evaluated.

### Statistics

All analyses were conducted in SPSS Statistics, version 26.0 (SPSS, Chicago, IL). Categorical data are presented as *n* and percentages. Continuous variables are presented as means with standard deviation. Chi-Squared tests, Fisher’s exact tests and independent *t* tests were performed as appropriate to compare subgroups of patients. Univariate analysis was performed to determine predictors of reporting of LLNs. Variables were selected a-priori based on assumed association with reporting, and included stage (cT3N0, cT3N+/T4), mesorectal N-stage (cN0, cN1, cN2), hospital type (academic, teaching or non-teaching), annual volume of rectal resections (high 60+, medium 30–59, low 0–29), threatened mesorectal fascia (≤ 1 mm distance) and height of the tumour (0–4 cm, 4–8 cm). Overall LR and lateral LR (LLR) were analysed for subgroups of patients with enlarged LLNs using Kaplan–Meier analysis and compared via log-rank test. A *p* value of ≤ 0.05 was considered statistically significant.

## Results

Of the 3057 patients included in this Snapshot study, 1096 patients were eligible for inclusion in the current study (Fig. 1). The mean age was 72.6 years and 66% were males. The median field of view was 220 mm (interquartile range (IQR) 200–240 mm) and no patients required exclusion due to an insufficient field of view of the lateral compartments. In total, 85% of patients also had a DWI series and the median slice thickness was 3 mm (IQR 3–4). Additional baseline characteristics are presented in Table 1.

**Table 1:** Baseline characteristics

*N* = 1096	*N* (%)
Gender: male (%)	719 (65.6)
Age in years (mean, SD)	72.6 (10.9)
Previous pelvic surgery (%)	95 (8.7)
Mean height of tumour from anorectal junction, cm (SD)	3.5 (2.5)
**Clinical T-stage (%)**	
cT3 total	943 (86.0)
cT3a	259 (23.6)
cT3b	373 (34.0)
cT3c	257 (23.4)
cT3d	54 (4.9)
cT4 total	153 (14.0)
cT4a	69 (6.3)
cT4b	84 (7.7)
**Clinical N-stage (%)**	
N0	324 (29.5)
N1	442 (40.3)
N2	331 (30.2)
**Positive mesorectal fascia (%) (distance ≤ 1 mm)**	
Yes	463 (42.2)
No	634 (57.8)
**Neoadjuvant radiotherapy (%)**	
None	222 (20.3)
Short-course radiotherapy	353 (32.2)
Chemoradiotherapy	519 (47.4)
Chemotherapy alone	2 (0.2)
**Type of primary operation (%)**	
Local excision	15 (1.4)
Anterior resection/partial mesorectal excision	35 (3.2)
Low anterior resection/total mesorectal excision	663 (60.4)
Abdominal perineal resection	373 (34.0)
Proctocolectomy	4 (0.4)
Other	7 (0.6)
**Resection margins**	
*R*0	1007 (91.9)
*R*1	89 (8.1)

### Original MRI reports

**Fig. 2:**
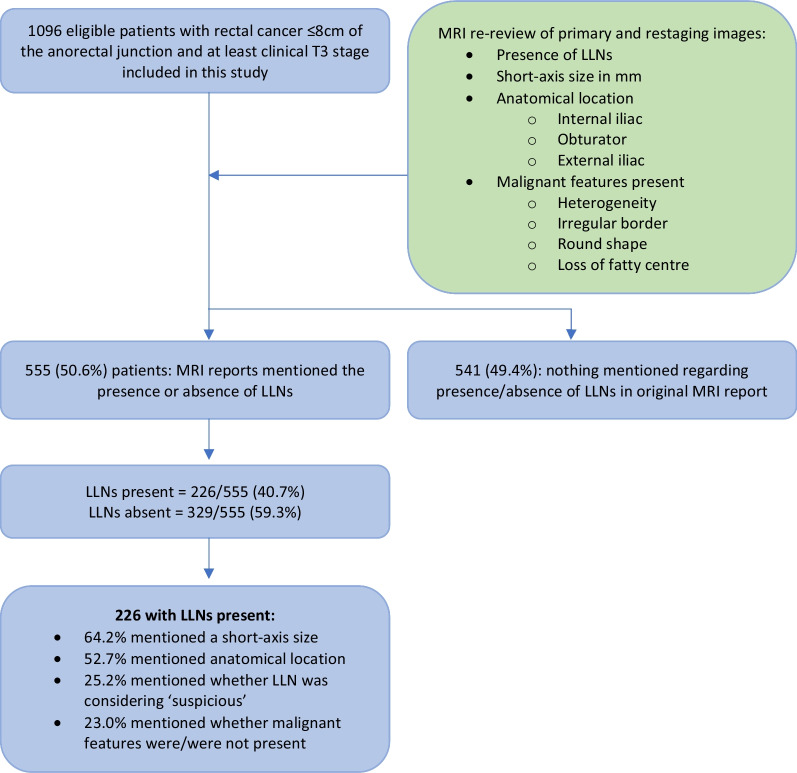
Flowchart of results according to MRI re-review

In 541 patients (49.4%), nothing regarding the presence or absence of LLNs was mentioned in the original MRI report. For the remaining 555 patients, the presence (226 patients, 40.7%) or absence (329 patients, 59.3%) of LLNs was explicitly stated. For those 226 patients with reportedly present LLNs, no additional characteristics were described in 35 patients (15.5%), and at least one characteristic was provided for the remaining 191 patients: short-axis size in 64.2%, anatomical location in 52.7%, suspiciousness in 25.2% and the presence or absence of malignant features in 23.0% (Fig. 2). An overview of all potential combinations of reported characteristics is shown in Table 2. For only two patients (0.9%), all characteristics were reported.

**Table 2:** Description of LLNs in primary MRI reports

Primary MRI reports and overall score	*N* (%)
Presence or absence of LLN mentioned	555/1096 (50.6)
Present	226/555 (40.7)
Absent	329/555 (59.3)
***Features described for present LLNs***	
Short-axis (SA) size mentioned	145/226 (64.2)
Compartment/anatomical location mentioned	119/226 (52.7)
Malignant features mentioned	52/226 (23.0)
Suspiciousness mentioned	57/226 (25.2)

Feature(s)	*N* (%)	*Examples of text*
MRI reports with mentioned LLN (*N* = 226)		
No features	35 (15.5)	*An extra-mesorectal lymph node*
Only SA node size	39 (17.3)	*7mm extra-mesorectal lymph node*
Only location	28 (12.4)	*Lymph node in the left internal iliac area*
Only malignant features	2 (0.9)	*Irregular lateral lymph node present*
Only suspiciousness	4 (1.8)	*Suspicious lateral lymph node*
SA node size and location	39 (17.3)	*7mm lymph node in the left internal iliac area*
SA node size and malignant features	7 (3.1)	*7mm heterogeneous lymph node*
SA node size and suspiciousness	19 (8.4)	*Suspicious 10-mm extra-mesorectal lymph node*
Location and malignant features	3 (1.3)	*Heterogeneous lymph node in left internal iliac area*
Location and suspiciousness	8 (3.5)	*Suspicious lymph node in internal iliac area*
Malignant features and suspiciousness	1 (0.4)	*Suspicious heterogenous extra-mesorectal lymph node*
SA node size, location and malignant features	16 (7.1)	*7mm heterogenous lymph node in the internal iliac area*
SA node size, location and suspiciousness	23 (10.2)	*Suspicious 7mm lymph node in the internal iliac area*
SA node size, location, malignant features and suspiciousness	2 (0.9)	*Suspicious heterogeneous lymph node of 7mm in the internal iliac area*

**Fig. 3:**
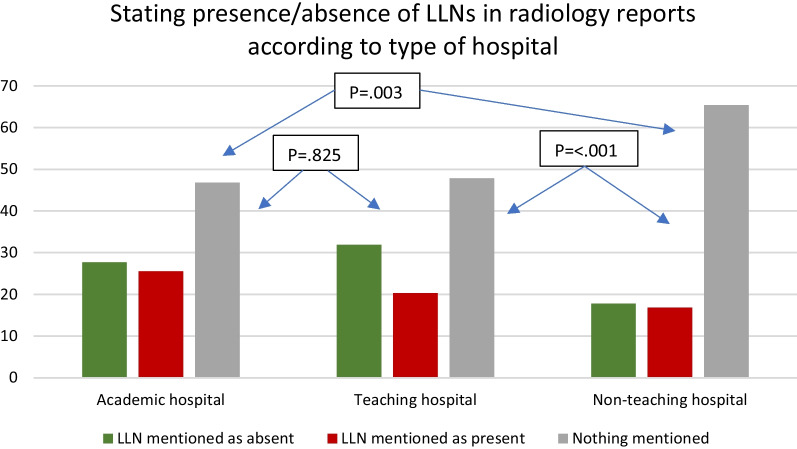
Stating the presence or absence of LLNs in primary MRI reports according to the type of hospital: academic, teaching or non-teaching

**Fig. 4:**
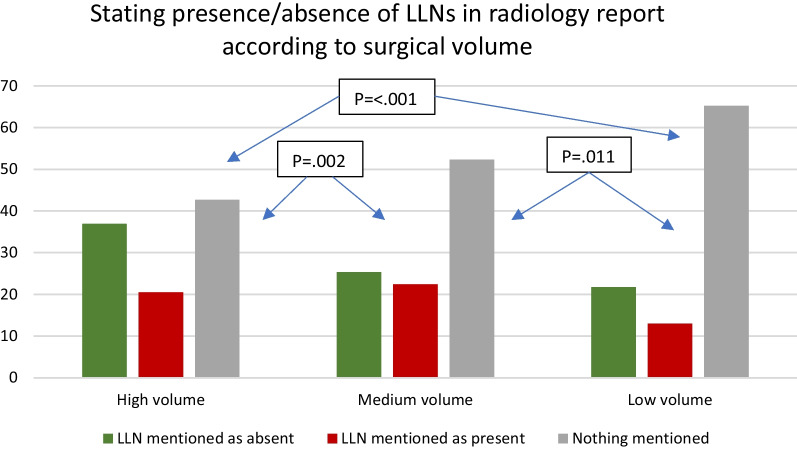
Stating the presence or absence of LLNs in primary MRI reports according to surgical volume of rectal resections per year. High volume: 60 rectal resections or more, medium volume: 30–59 resections, low volume: 0–29 resections (based on the number of resections according to part 1 of this Snapshot study for 2016)

Reporting LLNs was influenced by a number of factors. Reporting increased with higher stage (cT3N0 44.3% vs. cT3N+/T4 52.9%, *p* = 0.013) and when considering N-category based only on mesorectal nodes (cN0 44.1%, cN1 48.6%, cN2 59.5%, (*p* < 0.001)) with significant differences in subgroup analyses between cN1 and cN2 (*p* = 0.003) as well as cN0 and cN2 (*p* < 0.001), but not between cN0 and cN1 (*p* = 0.218). Additionally, both hospital type and volume of rectal resections significantly influenced reporting rates. Academic/teaching hospitals had significantly higher rates of reporting LLNs compared to non-teaching hospitals (34.6% non-teaching, 52.2% teaching, 53.2% academic: *p* = 0.006) while high/medium volume-centres outperformed low-volume centres (34.8% low, 47.8% medium, 57.3% high: *p* < 0.001). Importantly, 72% of non-teaching hospitals were also low-volume centres. Subgroup analyses revealed significant differences between the non-teaching hospitals versus academic/non-teaching (*p* = 0.003, *p* =  < 0.001, respectively) and between all three volumes (high vs. medium, *p* = 0.002, high vs. low *p* < 0.001, medium vs. low, *p* = 0.011) (see Figs. 3, 4). Reporting LLNs was not significantly influenced by a threatened mesorectal fascia (*p* = 0.926) or distance of the tumour from the anorectal junction (*p* = 0.597).

### MRI re-review

MRI re-review by the additionally trained abdominal radiologist(s) per participating centre, resulted in 379 patients (34.6%) with visible LLNs and 139 patients (12.7%) with an enlarged LLN (short-axis ≥ 7 mm).

**Fig. 5:**
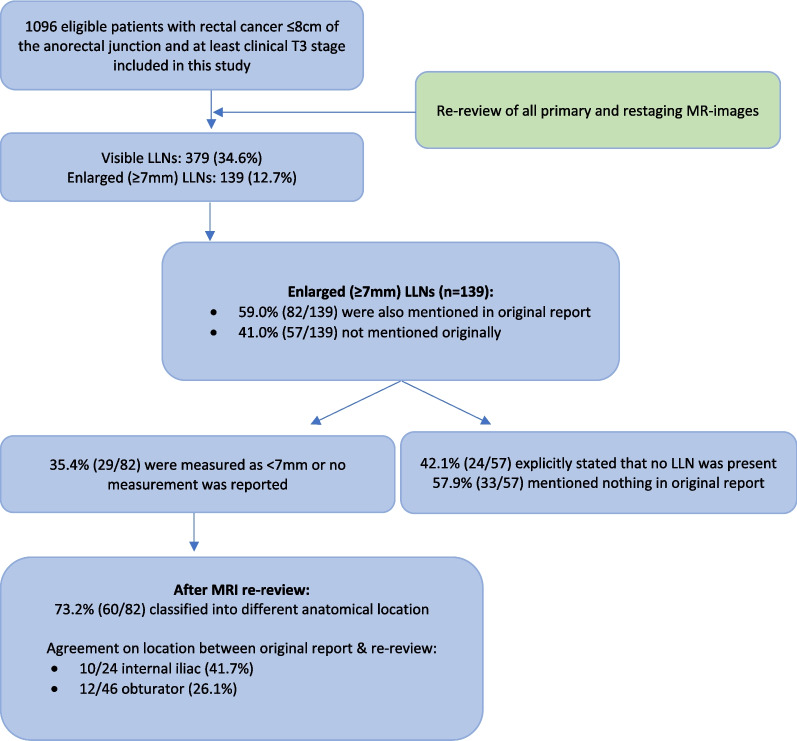
Flowchart of results for enlarged LLNs according to MRI re-review

For these 139 patients with enlarged LLNs, the presence of an LLN was originally mentioned in 82 cases (59.0%). In 29 of these 82 cases (35.4%), LLNs were originally measured to be < 7 mm or no size was mentioned. For the remaining 57 out of 139 patients (41.0%) with enlarged LLNs identified during MRI re-review, LLNs were not mentioned in the primary MRI report: 24/57 cases (42.1%) explicitly stated that no LLN was present and nothing was mentioned for 33/57 cases (57.9%) (Fig. 5).

For the 82 cases in which LLNs were mentioned in the reports and during re-review, 73.2% (60/82) were classified into a different anatomical location during re-review, including 40 patients for whom no location was originally mentioned. Agreement between original reports and re-review concerning location was found for 10 of the 24 internal iliac cases (41.7%) and for 12 of the 46 obturator cases (26.1%). For the same 82 cases, these LLNs were significantly more often located in the internal iliac compartment (24/82 (29.3%) vs. 10/57 (17.5%) *p* = 0.001) and had a larger mean size (10.1 mm (7–20 mm) vs. mean 8.5 mm (7–19 mm) *p* = 0.002) compared to those not mentioned in the original reports.

### (Lateral) locoregional recurrence rates

**Fig. 6:**
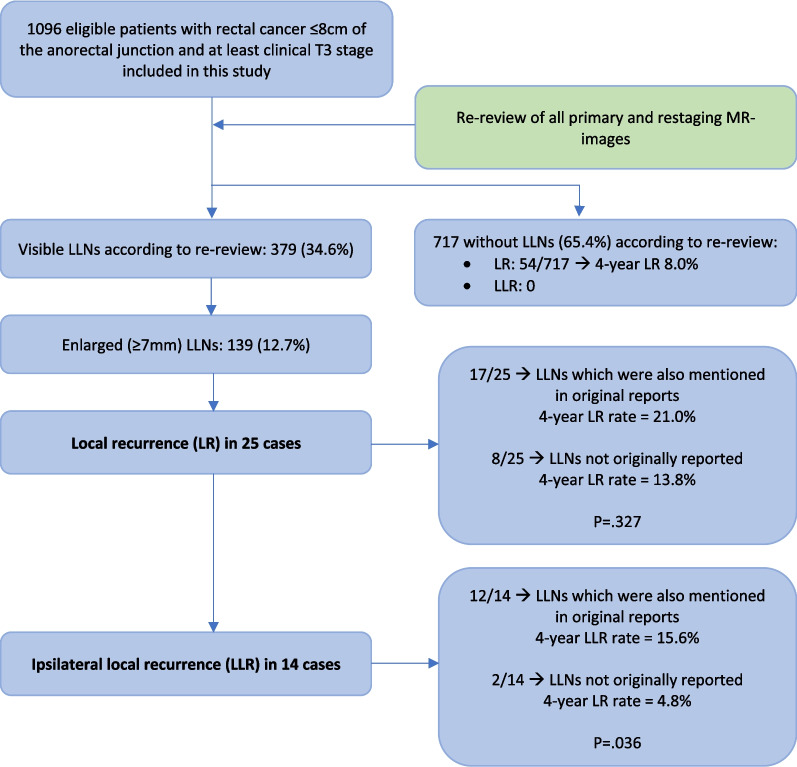
Flowchart of long-term oncological results

The median follow-up was 48 months (IQR 32–55). Overall, 102 patients developed an LR and 22 patients an LLR, with 4-year rates of 10% and 2.3%, respectively (Fig. 6).

Of the 139 patients with enlarged LLNs identified during MRI review, an LR occurred in 25 patients (17.9%); 17/82 patients (20.7%) where LLNs were also mentioned originally, and 8/57 patients (14.0%) where LLNs were not originally reported. The 4-year LR rates were 21.0% vs. 13.8%, respectively (*p* = 0.327). In total, 22 (88.0%) received neoadjuvant radiotherapy and the mean LLN size was 9.7 mm (SD 3.4): 7.6 mm for those not mentioned originally compared to 10.3 mm mentioned in both reports. Positive resection margin rates were not significantly different among those who developed an LR per group (23.5% [4/17] versus 14.3% [1/8], respectively, *p* = 0.612).

An LLR occurred in 14 patients (10.1%), 12/82 patients (14.6%) with LLNs ≥ 7 mm mentioned originally and during MRI review and in 2/57 patients (3.5%) identified only during the review. The 4-year LLR rates were 15.6% and 4.8%, respectively (*p* = 0.036). In total, 13 (92.9%) received neoadjuvant radiotherapy. The mean LLN size was 9.6 mm (SD 3.4); 9.8 mm for those mentioned in both reports versus 8.2 mm for those not mentioned originally. Two patients with LLR also had positive resection margins, one case described LLNs originally and during re-review and one case where LLNs were only identified during re-review (*p* = 0.119).

For the 717 patients who according to the review did not have LLNs, 54 (7.5%) developed an LR (4-yr LR rate 8.0%) and none developed an LLR.

## Discussion

This national, cross-sectional retrospective cohort study found that in 49.5% of cases, primary MRI reports of patients treated for cT3-4 rectal cancer located ≤ 8 cm from the ARJ in 2016 in the Netherlands did not mention the presence or absence of LLNs. Non-reporting of LLNs was highest when considering non-teaching (65.4%) or low-volume rectal resection centres (64.7%). Most importantly, 41% of all enlarged LLNs found during MRI re-review were not mentioned in the original MRI reports. Our results indicate a significant lack of awareness for LLNs during routine daily practice and knowledge regarding their importance for treatment decisions and the risk for LR development. The current results also indicate a benefit of training.

An important finding in this population-based study is the difference in the identification of LLNs between the type/volume of the hospital. The lowest rates of reporting were found for low-volume, non-teaching hospitals, though it is important to note that 72% of non-teaching hospitals were also low-volume centres, highlighting the heterogeneity in knowledge, possibly due to limited exposure. However, the highest rate of reporting, found for high-volume centres, was still limited to 57.3%. These results advocate for significant improvement. One method is the introduction of structured radiology templates at a national level with incorporation in guidelines. Numerous studies have proven that templates significantly improve the reporting of specific items [27–31] and considering the results found here, templates with targeted learning would promote homogeneity and clarity [32, 33]. Also, the establishment of thorough guidelines should help assure appropriate reporting of all features relevant to rectal cancer cases.

The literature has shown that an LLN short-axis size of ≥ 7 mm increases the LR rate to almost 20% [15]. However, for 41% of patients with enlarged LLNs identified during re-review, LLNs were not mentioned in the original reports. A further 35% of LLNs would have been classified into a different size category (</≥ 7 mm) after re-review. Considering the significance of short-axis size for treatment planning and oncological outcomes [8, 9, 16–18, 34], these rates are insufficient. However, the oncological results found in the present study are reassuring, despite the ‘missed’ nodes. Only two LLRs (4.8% 4-year LLR) occurred in the group with enlarged LLNs which were ‘missed’ during original reporting. This low number may be due to the fact that 49 of these 57 patients (86%) received some kind of neoadjuvant radiotherapy, which will likely have covered the LLNs to some extent. Considering that many centres will have used 3D-conformal/box techniques for irradiation in 2016, these wider margins may have allowed for the inclusion of LLNs. This may change in the future as more conformal techniques will be applied, resulting in a steeper dose fall-out for unidentified LLN areas which are not incorporated in the clinical target volume. Additionally, with improved image-guided radiotherapy, margins will decrease to limit toxicity, potentially reducing the dose even further to any unidentified LLNs.

For LLNs identified in both the original reports and during re-review, a high 4-year LLR rate of 15.6% was found. It is possible that this is the result of the under-treatment of enlarged LLNs. Despite adequate recognition and assumed adequate radiotherapy, the LLNs still resulted in recurrent disease. A lateral lymph node dissection (LLND) might have lowered these rates, for which evidence showed a reduction in LLR rates for internal iliac LLNs from 52 to 8.7% after LLND [15]. The oncological benefits of such a procedure should be considered for patients with enlarged LLNs. To further investigate the specific role of radiotherapy, part 3 of this Snapshot study will analyse individual radiotherapy delineation volumes to ascertain the doses that LLNs received and whether they were adequately positioned in the irradiation fields. If this reveals LLNs to already adequately receive > 95% of irradiation doses, then it is possible that higher LLR rates may only be able to be tackled with LLND surgery.

The importance of LLN location has been suggested in the recent literature [16]. The current study found significant discrepancies between the locations mentioned in primary MRI reports compared to re-review after dedicated training, with a 73.2% change in compartment classification. Clear definitions were used to determine the lateral compartments [16], which may rationalise the results found here. In the absence of an (inter)national guideline stating explicit borders, many original reports were probably based on personal experience and/or preferences. Considering the vast changes in the location found here after adhering to one classification, the oncological implications of these locations need to be analysed in the future.

While the population-based cross-sectional design of this study, with trained radiologists, carries an important strength to evaluate the national awareness of LLNs, there are also limitations that need to be discussed. Primarily, this study can only establish whether LLNs were mentioned in reports, which is not a perfect translation of awareness. There were also no restaging reports or MDT meeting reports available, preventing the investigation of whether LLNs were discussed there. Seven of the 67 hospitals performing rectal cancer resections in the Netherlands did not participate, and these were all non-teaching, low-volume centres. This might have influenced the current results, with potentially even higher non-reporting rates. Furthermore, there was also heterogeneity present within the patient population concerning differences in neoadjuvant and surgical treatments and no data regarding MRI resolution was available. Lastly, the original reports are from 2016 due to the 4-year oncological follow-up period. It is very possible that a present-day repeat of this study would find improved results considering the increase in the literature and research concerning LLNs.

## Conclusion

This national, cross-sectional, retrospective cohort study found that in almost half of primary MRI reports for patients with advanced rectal cancer, LLNs were not mentioned. Furthermore, a significant proportion of enlarged LLNs found during re-review were not mentioned in the original reports, which may have influenced treatment outcomes. These results highlight the need to increase awareness of LLNs and the implementation of structured templates for MRI reports to include a dedicated section for LLNs.

## Appendix A

Terms accepted as referring to LLNs:

- Extra-mesorectal
- Lateral lymph nodes
- (Lymph nodes) in obturator area
- (Lymph nodes) in iliac area
- Para-iliac
- Para-obturator
- (Lymph node) along internal iliac artery
- (Lymph node) along external iliac artery
- (Lymph node) along obturator artery
- Outside the mesorectal fat
- Outside the mesorectal fascia
- Outside the mesorectum

Terms such as

- Lymph nodes
- Nodes

without any accompanying information were considered as ambiguous and not clearly referring to LLNs.

## Abbreviations

LLND: Lateral lymph node dissection
LLNs: Lateral lymph nodes
LLR: Lateral local recurrence
LR: Local recurrence
MRI: Magnetic resonance imaging
TME: Total mesorectal excision
